# Next-Generation *in vivo* Modeling of Human Cancers

**DOI:** 10.3389/fonc.2018.00429

**Published:** 2018-10-10

**Authors:** Gaetano Gargiulo

**Affiliations:** Molecular Oncology, Max Delbrück Center for Molecular Medicine, Berlin, Germany

**Keywords:** mouse models, cancer, single-cell genomics, CRISPR/Cas9, targeted therapies, biomarker discovery, preclinical models, patient-derived xenografts (PDX)

## Abstract

Animal models of human cancers played a major role in our current understanding of tumor biology. In pre-clinical oncology, animal models empowered drug target and biomarker discovery and validation. In turn, this resulted in improved care for cancer patients. In the quest for understanding and treating a diverse spectrum of cancer types, technological breakthroughs in genetic engineering and single cell “omics” offer tremendous potential to enhance the informative value of pre-clinical models. Here, I review the state-of-the-art in modeling human cancers with focus on animal models for human malignant gliomas. The review highlights the use of glioma models in dissecting mechanisms of tumor initiation, in the retrospective identification of tumor cell-of-origin, in understanding tumor heterogeneity and in testing the potential of immuno-oncology. I build on the deep review of glioma models as a basis for a more general discussion of the potential ways in which transformative technologies may shape the next-generation of pre-clinical models. I argue that refining animal models along the proposed lines will benefit the success rate of translation for pre-clinical research in oncology.

## Introduction

Modeling human tumors in animals has been the leading approach to translational research in oncology over the last three decades. Successes in the field include, among others, identifying druggable targets for aggressive subtypes of leukemia, breast cancer and melanoma. In the late 1990s, the retinoic acid (RA) was successful to induce full remission in 71–91% of patients with acute promyelocytic leukemia (APL) in clinical trials that compared this treatment with standard chemotherapy (1). The first transgenic murine models for human APL were generated by inserting the oncogene *PML-RAR* in promyelocytes downstream the Cathepsin G or *hMPR8* regulatory elements (2, 3). These models showed sensitivity to RA (4) confirming their value in the treatment of APL and making them the leading models to investigate responses to anti-cancer treatments at the cellular and molecular levels.

In solid tumors, Trastuzumab (clinical name Herceptin) was approved for treatment in Her2 positive breast cancer. Trastuzumab is an antibody binding to the EGF receptor Her2 and clinical trials showed benefit in Her2 positive breast cancer patients in terms of progression-free and overall survival (5). When the murine p185 antibody was tested in murine breast cancer xenograft models, it proved to be effective in counteracting tumor growth (6). Shortly afterwards, the murine Her2 antibody was humanized to allow its use in clinical trials (7).

Kinase inhibitors were considered the poster child of targeted therapies in the early 2000s and several were approved for treating different malignancies. Imatinib (clinical name, Gleevec) was approved to block the signaling activity of the BCR-ABL fusion protein oncogene in Chronic myeloid leukemia (CML) (8). In mice, the dependence for CML cells on BCR-ABL and the main features of response and resistance to Imatinib could be successfully demonstrated (9). In addition to mouse models, larger animal models such a spontaneous Canine B-cell Lymphoma have also been used to validate the therapeutic outlook for kinase inhibitors (10). The Ibrutinib, a Bruton's tyrosine kinase (BTK) inhibitor validated in this way, later showed a durable efficacy in relapsed or refractory mantle cell lymphoma patients as single agent (11), or in combination with an anti-CD20 antibody (clinical name, Rituximab) (12), which was later extended to chronic lymphocytic leukemia (13).

In the case of the specific BRAF^V600E^ inhibitor Vemurafinib (14), preclinical models not only validated the response to BRAF inhibition but also revealed the RAF paradox, meaning that ERK signaling is amplified by RAF inhibition in *BRAF*-wild-type, *RAS* mutant tumors, despite RAF's position downstream RAS (15). In Non-Small Cell Lung cancer (NSCLC), Crizotinib showed great anti-tumor potential against EML4-ALK fusion positive carcinomas, and it has quickly gained momentum for treating a subset of NSCLC patients (16), after successful evaluation in a transgenic mouse model for lung adenocarcinoma (17).

Despite the great success of those compounds, which benefits many cancer patients, targeted therapies are accompanied by acquired resistance, when cancer cells experience gatekeeper mutations or activate alternative pro-tumorigenic pathways (18, 19). To overcome this limitation, targeted therapies are combined with standard chemotherapy or combinations of targeted therapies are sought in appropriate pre-clinical settings (20).

In the clinics, drug combinations often aim at extending on-target toxicity by targeting the same mechanisms (i.e., unrestricted proliferation) with multiple drugs. For instance, the combination of four different chemotherapeutic agents Doxorubicin, Bleomycin, Vinblastine, and Dacarbazine (ABVD) is the standard of care in the treatment of Hodgkin lymphoma (21). However, the use of multiple drugs is accompanied by the increased burden of side effects for patients. Testing more sophisticated approaches, such as synthetic lethality in animal models represents therefore the ideal pre-clinical development. Synthetic lethality is a concept originated from yeast biology and reflects the observation that simultaneously hitting two closely related mechanisms can lead to significant toxicity, whereas single treatments are largely well-tolerated. This offers the possibility to target cancer cells bearing specific alterations, sparing normal cells from side effects. The paradigm of a synthetic-lethal treatment that has made its way into clinical application is the use of Poly-(ADP-ribose) polymerase (PARP) inhibitors in ovarian cancer and breast cancer (22). Targeting PARP proved to be synthetic lethal with concurrent alterations in homologous recombination (HR) DNA repair genes, such as *BRCA1* and *BRCA2*. In pre-clinical research, *BRCA*-mutant mouse models have been instrumental in highlighting the strength of these treatments and have illustrated potential ways to circumvent their limitations (23). Clinical trials have led to the FDA approval of a number of PARP inhibitors showing efficacy in treatments for ovarian, breast and prostate cancer. In a BRCA-competent triple negative breast cancer/TNBC) DDR-mediated PARP antitumor activity was reinforced by concurrent PI3K-AKT-mTor pathway inhibition (24). In general, predicting long-term responders to PARP inhibitors is a critical issue that has yet to be solved by future pre-clinical breakthroughs.

In addition to their roles as discovery platforms, accurate pre-clinical models can also predict patients' response to a given treatment. In a seminal study, Singh et al. retrospectively assessed targeted therapies either alone or in combination with standard-of-care treatments thereby replicating large-scale human clinical trials (25). This was achieved by applying a similar treatment protocol and evaluating the clinical endpoints overall survival (OS) and progression free survival (PFS) for *in vivo* studies. Animal models for pancreatic ductal adenocarcinoma (PDAC) and non-small cell lung cancer (NSCLC) were exploited as surrogate for KRAS^*G12D*^ patients' response to Erlotinib and Bevacizumab, therapeutics targeting epidermal growth factor (EGFR) and vascular endothelial growth factor (VEGF), respectively. The standard of care (Carboplatin or Gemcitabine) was combined with targeted drugs thereby mimicking the original studies (25). Retrospectively, this post-clinical study obtained (largely negative) results comparable to those for human clinical trials. This set an excellent standard for future design of pre-clinical studies.

Currently, technological breakthrough in the field of genetic engineering and single cell genomics are enabling us to create ever-more sophisticated animal models of human cancers, and to exploit them in achieving a better translation of pre-clinical studies. Here, I focus on animal models for human malignant gliomas as an entry point for a retrospective review of the use of animal models in tumor biology and therapy. Thereafter, I review state-of-the-art technologies and offer future perspective to incorporating these in generating and exploiting animal models as pre-clinical tools for cancer biology and intervention, which can be valid for glioma and more in general for different types of human cancer.

## Experimental models for high-grade gliomas

### Autochthonous mouse models

Autochthonous mouse models for human cancers are obtained by initiating tumors in a normal cell *de novo* and within the intact organism. The main advantage of these models is the pathophysiological relevance of the tumor initiation.

Mouse models for Glioblastoma Multiforme (GBM) have been systematically used to investigate tumor initiation and progression in the context of a living organism. The first example of genetically engineered mouse models (GEMMs) in modeling gliomas was developed in the Aguzzi lab. It was generated through the over-expression of the *v-Src* oncogene via the glial fibrillary acidic protein (GFAP) regulatory elements (26). Later, Holland and Varmus introduced to the scientific community a model based on avian retroviral gene transfer, the RCAS-TVA, which still counts as one of the most commonly used models for gliomas (27).

By far, the largest use for autochthonous models in GBM has been in the systematic dissection of the mechanisms leading to tumor initiation. Several studies have addressed the role of signaling pathways, validated the genetic dependencies on individual genes, and investigated the contribution of non-cell autonomous factors in GBM initiation (Table 1). These studies have uncovered critical pathways in tumor initiation and subsequent genetic aberrations found in high-grade lesions. The unifying conclusion from gliomagenesis in autochthonous models is that GBM is primarily driven by an intricate mix of pro-oncogenic hits cooperating with inactivation of tumor suppressive pathways. On the one hand, hyper-activation of the AKT and MAPK pathways has to occur through either supra-physiological receptor tyrosine kinase (RTK) activity (e.g., EGFR, PDGFRA) or a loss of negative regulators, such as PTEN and NF1. On the other hand, the Rb and p53 pathways must be circumvented by either direct inactivation or through deletion of the *INK4A*/*ARF* locus (also known as *CDKN2A*).

**Table 1:** Autochthonous mouse models for glioma.

**Genetic alterations**	**Cell type specificity**	**Potential cell of origin**	**Selected conclusion(s)**	**References**
*v-Src* (gain)	GFAP	Astrocytes	Glioma formation driven by *v-Src* expression in astrocytes	Weissenberger et al. (26)
*EGFRvIII* (gain); *p16, p19* (loss)	GFAP, Nestin	Astrocytes and NPCs	Patient-specific alterations are capable of transformation; NPCs are more prone to transformation	Holland et al. (27)
*AKT-Myr Δ11–60 KRAS^*G*12*D*^*	Nestin	NPCs	RAS and AKT signaling cooperatively but not exclusively are capable of transforming NPCs.	Holland et al., (28)
*NF1, Trp53* (loss)	ubiquitous	Astrocytes and NPCs	Spontaneous gliomagenesis in animals with tumor suppressors deficient background; role for genotype in spontaneous tumor formation.	Reilly et al. (29)
*GFAP-HRAS^*G*12*V*^*	GFAP	Astrocytes	Supraphysiological RAS activation in astrocytes can lead to pathologically-relevant alterations	Ding et al., (30)
*v-erbB* (*EGFR*; gain); *Trp53, p16, p19* (loss)	S100B	Glial cells	Overexpression of *v-erbB* (EGFR) induces oligodendroglioma and GBM in combination with *p53* or *CDKN2A* deletion; spontaneous loss of chromosomal DNA synthenic to human chromosome 1p	Weiss et al., (31)
*NF1* (loss)	GFAP KO+ubiquitous HET	Astrocytes	spontaneous optic nerve glioma formation in animals in which *NF1* is biallelically deleted in astrocytes as well as heterozygously inactivated in the microenvironment	Bajenaru et al., (32)
*NF1, Trp53* (loss)	GFAP	NPCs	Complete penetrance for *NF1* and *p53* mutant gliomas; evidence for NSC as cell of origin for gliomas	Zhu et al., (33)
*NF1, Trp53, PTEN* (loss)	GFAP	NPCs	*AKT* activation driven by *PTEN* loss coorelated with GBM grading in the mouse tumors	Kwon et al., (34)
*v-erbB* (*EGFR*); *Trp53* KO	S100B	Astrocytes and OPCs	Evidence for Side-population cancer stem-like cells in a mouse model for oligodentroglioma	Harris et al., (35)
*hPDGF-B*	GFAP	Astrocytes and spinal cord	Tet-inducible model for spinal oligoastrocytoma	Hitoshi et al., (36)
*PDGF-B-IRESβGeo*; *p53*^−/−^;	GFAP	Astrocytes	*PDGF* stimulation and *p53* loss induce tumors in diferent parts of the brain	Hede et al., (37)
*RB, p53, PTEN*	GFAP	NPCs	Inactivation of different combinations of tumor suppressor genes in SVZ causes brain tumors with different phenotypes	Jacques et al. (38)
*NF1, Trp53* (loss)	GFAP, Nestin or NG2	NPCs and OPCs	OPCs can serve as cell of origin for gliomas	Liu et al., (39)
*PTEN, p53*	retroviral activation	NPCs	*PDGF* stimulation and deletion of *PTEN* and p53 lead to a Proneural-like expression phenotype	Lei et al., (40)
*HRAS^*V*12^*	lentiviral GFAP activation	Astrocytes and NPCs	Gliomagenesis is more effective in the hippocampus and the subventricular zone than in the cortex	Marumoto et al., (41)
*INK4A/ARF; KRAS^*v*12^PTEN; INK4A/ARF; KRAS^*v*12^ p53; INK4A/ARF; KRAS^*v*12^*	lentiviral CMV or GFAP activation	Astrocytes and NPCs	Higher penetrance and faster gliomagenesis in CMV- vs. GFAP-lenti-Cre activated mutations.	de Vries et al., (42)
*PTEN, TP53*, and *RB1*	GFAP	Astrocytes and NPCs	*PTEN* or *RB* deletion drive somatic amplifications of genes in the PI3K or Rb pathways	Chow et al. (43)
*NF1, Trp53* (loss)	SynI-Cre, GFAP-Cre, Nes-Cre	Neurons, Astrocytes and NPCs	Neurons can be transformed by delivery of shRNAs targeting *NF1* and *p53*. Dedifferentiation toward NPCs is pbserved in targeted matureastrocytes.	Friedmann Morvinski et al. (44)
*p53, PTEN, NF1*	Nestin	NSCs	CSCs can exploit quiescence similar to adult neural stem cells (NSCs) to contribute to relapse after chemotherapy.	
*KRAS* and *AKT* or *PDGFB* in *ARF*^−/−^ and *INK4A/ARF*^−/−^	CNP+ SVZ	OPCs	*KRAS* & *AKT* or *PDGFB* dictates astrocytic or oligodendroglial tumor development from OPCs	Lindberg et al. (45)
*p53, PTEN, NF1*	Ascl1 or Ng2	Astrocytes, NPCs, or OPCs	The cell of origin emerges as a major determinant of GBM molecular subtype	Alcantara Llaguno et al. (46)
*p53, PTEN, NF1*	electroporation delivery	NPCs	Cas9 and sgRNAs delivered to the cerebral verntricular zone lead to transformation	Zuckermann et al. (47)
*RBP-JK, p53*	Hes5	NPCs	Notch signaling is tumor suppressive and contributes to the formation of primitive neuroectodermal-like lesions	Giachino et al. (48)
BCAN-NTRK1 fusion protein	adenoviral delivery	NPCs	Expression of the EML4-ALK fusion protein drives gliomagenesis	Cook et al. (49)
56 (brain) tumor suppressors	adenoviral delivery	astrocytes	*PTEN* mutations enhances resistance to therapy in *RB1* mutated glioma	Chow et al. (50)
*PDGF-B; ARF^−/^*^−^	GFAP+ SVZ, CNP+ SVZ, NES+ cortex progenitors	NSCs, OPCs, NPCs	A neural stem-cell-like origin produces higher malignancy and drug sensitivity	Jiang et al. (51)
*BCAN-NTRK*; *MYB-QK*; *BRAF^*V*600*E*^*	GFAP or NES	astrocytes or NPCs	A diverse set of CRISPR-mediated genomic alterantions lead to tumorigenesis	Oldrini et al. (52)

The cellular origin of the disease is an additional intense area of research enabled by GEMM models. Mouse models are useful in this endeavor because the retrospective nature of this assessment makes it hard to precisely identify the cellular origin of cancer in humans. The identification of metastable GBM molecular subtypes and genetic biomarkers, however, provided indirect evidence that fully fledged tumors potentially bear a signature of their potential cell-of-origin (53, 54). This has been now extended to several other cancers (55). Importantly, data in GEMMs are consistent with this view. Overall, from a formal literature review it emerged that: (i) different mutations appear to dictate the cellular phenotype of the resulting tumors; (ii) tumors with similar alterations but originating in different cellular compartments have private biological properties (38, 43, 45, 46, 48, 56). Mouse models have produced overwhelming evidence that undifferentiated neural stem and progenitor cells can efficiently serve as cell-of-origin for the disease in the experimental models (Table 1). Yet it is also evident that nearly every cell in the mouse brain, including post-mitotic neurons, has a potential for transformation, if the oncogenic pressure is significant enough (44). While these studies do not necessarily address the pathophysiological relevance of the models for the origins of human gliomas, two strong messages have emerged from this research. First, neural stem cells are significantly more prone to transformation than the differentiated cells composing the brain parenchyma. Consistently, sequencing of the human SVZ appears to suggest that pre-transformation clones tend to reside in the area of the human brain containing the most undifferentiated neural progenitors (57). Second, regardless of the cell targeted by the oncogenic signaling, the formation of a glial-like malignant progenitor population appears to be an obligatory step in the malignant transformation.

Despite the sophisticated and elegant approaches that were used to generate GEMMs for glioma, the implications of these findings for therapy have so far been limited. Cell cycle regulation emerged as a major predictor of therapeutic response (51, 58), a finding that does not offer additional therapeutic options *per se*, but represents important ground for future glioma modeling. As discussed below, these studies provide enhanced confidence in transplantation models generated by transforming normal primary cells.

Recently, improved genetic engineering delivery and effectors, such as the CRISPR/Cas9 system have opened new routes to modeling human tumors, including gliomas. CRISPR/Cas9 operates via either non-homologous end joining (NHEJ) that generates genome deletions and insertions (indels), or by homology directed repair (HDR). This approach permits a direct genetic engineering of endogenous loci thereby avoiding random genome integration and potential genotoxicity, which is the intrinsic risk associated with retroviral delivery. For instance, *in utero* electroporation of gRNAs and Cas9 allowed the simultaneous *in vivo* deletion of the *Trp53, Pten*, and *Nf1* tumor suppressor genes directly in the brain. This approach bypassed tedious modifications at zygote level (47). Considering that *Trp53* and *Nf1* are located in close proximity to each other in the genome, conventional breeding is unlikely to produce mutants through their separation onto different chromatids during chromosomal crossover without very time-consuming efforts. This illustrates one way for CRISPR/Cas9 to significantly speed up the pace at which disease models can be generated, including cancer (reviewed, among others, by Sanchez-Rivera and Jacks) (59). Most likely, other investigators will quickly adopt *in vivo* electroporation.

In addition to simplifying single gene modifications, CRISPR/Cas9 has permitted the modeling of complex karyotypes. For instance, CRISPR/Cas9 was instrumental to generate the mouse equivalent of fusion proteins previously discovered in human cancers (49). The intracranial adenoviral delivery of CRISPR/Cas9 directed microdeletions in the genomic loci of *Bcan* and *Ntrk1* (Brevican and Neurotrophic Receptor Tyrosine Kinase 1, respectively), led to the generation of a mouse model for glioma in which tumorigenesis is driven by BCAN-NTRK1 fusion. This model demonstrated a good response to the kinase inhibitor Entrectinib, which preferentially targets tropomyosin receptor kinases, including NTRK1 (49). This report by the Ventura lab builds on their own pioneering work in generating an EML4-ALK fusion model for NSCLC (60), using CRISPR/Cas9 to systematically screen for fusions as oncogenic drivers in solid tumors. Using this approach, in addition to the chromosomal deletion required for the *Bcan*-*Ntrk1* model, it has been possible to model the *Myb*-*Qk* chromosomal translocation as well as induce an equivalent of the human BRAF^V600E^ point mutation by homology-directed-repair (52). The latter improvements in genetic engineering now make easier to generate more complex genotypes in autochthonous models. For instance, mutations in the Isocitrate dehydrogenase 1 (IDH1) are associated with a number of blood and solid tumors, including GBM. While a IDH1^R132H^ models were generated before using classic transgenesis (61, 62), the ability of modifying single loci or create large chromosomal deletions paves the way to generate autochthonous models for human gliomas based on 1p/19q, *IDH*, and *TERT* Promoter Mutations, which represent specific entities in humans (63, 64).

Collectively, these studies support GEMMs as invaluable tools in exposing the principles underlying glioma genetics and biology. Non-autochthonous models (described below) have a different set of advantages but clearly GEMMs stand to gain even more momentum in the CRISPR/Cas9 era.

### Transplantation models for glioma

Transplantation models represent the most widely used alternative to GEMMs, and are essentially built using cells endowed with the ability to initiate tumors in secondary recipient animals. This modeling approach offers the flexibility of spatiotemporal control on tumor initiation and of the potential to experimentally manipulate individual cells. The impact of such manipulations, in turn, can be tested competitively during tumor growth or response to treatment.

The implantation can be carried out in either the tissue in which the disease originated (orthotopic), or more accessible locations, such as the flank of a recipient animal. The choice of the tumor cell and the recipient animal defines whether a model is to be considered syngeneic, homotypic, heterotypic or xenogeneic. For instance, the transplantation of GL261 mouse glioma cells in C57BL/6 recipient mice is considered syngeneic, because GL261 also have a C57BL/6 genetic background. If the background of the donor and recipient animas is not identical, the model is defined as homotypic. A transplantation mode is defined as xenotransplantation if the genotype of the donor is from humans, whereas it is heterotypic for genomes from every species other than humans. Transplantation-based models represent a trade-off between limitations on the pathophysiological relevance and an enhanced control over the temporal initiation of the disease as well as the unique feature of permitting perturbation experiments of various types. Since the first transplantation experiments in nude mice (65, 66), transplantation has permitted testing the tumorigenic and developmental potential of glioma, dissecting its heterogeneity and characterizing underlying molecular mechanisms. Given the flexibility and scalability of this system, it is the best choice for individual target discovery and validation as well as for developing disease-relevant systematic discovery platforms *in vivo*.

### Syngeneic transplantation models

Syngeneic tumor models have widely used GL261 cells, due to the fact that they exhibit key alterations in RAS, p53 and PI3K and other pathways which are commonly deregulated in human GBM. Tumors generated in this way share features with human GBM including the upregulation of VEGF and HIF-1α and a diffuse invasion pattern while retaining an intact immune system (67). Lately, syngeneic models have regained more attention with the increasing focus on cancer immunotherapy.

Evidence in cancer patients and mouse models have substantially supported that tumors can be immunogenic but also induce acquired immune tolerance (68). Thus, with the exception of tumors with high mutational and neoantigen load, such as melanoma and lung cancer (69), in heterogeneous solid tumors, the immune-checkpoint inhibitors are unlikely to be effective as single agents. Syngeneic models are well-positioned to evaluate the efficacy of combination therapies, which include the immunotherapy component. In gliomas, one viable combination therapy tested in syngeneic models is the efficacy of CAR T-cells (i.e., T-cells expressing chimeric antigen receptors) with the standard-of-care (70). In a transplantation setting, syngeneic splenocytes from C57BL/6 or VM/Dk were directed against GL-261 or SMA-497, SMA-540, and SMA-560 cells, respectively, by using the full-length NKG2D protein fused to CD3. This system has the advantage of targeting poorly expressed antigens, which is therefore better systemically tolerated. Moreover, NKG2D ligands are multiple antigens, therefore making it more difficult for the tumor to escape. Finally, NKG2D ligands expression appears to increase upon temozolomide and radiotherapy (70, 71), making these targets particularly attractive in an adjuvant setting. Future preclinical testing should include metalloproteases inhibitors, since *ADAM10* and *ADAM17* expression by tumor cells appear to provide a simple solution to immunevasion by producing soluble NKG2D (72), thereby dampening the γδ T-cell adaptive response (71).

Training the immune system against tumor cells using vaccines could potentially induce a long-term immune response. In a syngeneic mouse model, dendritic cell vaccination using glioma stem-like cells (GSCs) lysate resulted in a measurable response in mice (73). This approach, however, is still being perfected in the setting of human vaccination against brain tumors, as witnessed by clinical trials that failed to show an objective clinical response (74, 75).

Evidence from immunocompromised HIV/AIDS patients is compatible with the speculation that the adaptive immune system is most effective in controlling truly foreign antigens. In fact, HIV/AIDS patients largely develop virus-associated cancers with increased frequency but not other antigenically “colder” tumors (76). A compelling solution to selectively induce immune responses against foreign antigens in tumor cells is offered by oncolytic viruses (OVs). OVs with tropism for cancer cells can simultaneously act at different levels in the tumor microenvironment (TME). Local killing of tumor cells works as *in situ* vaccines, alerting antigen-presenting cells (APC) to multiple tumor-associated antigens (TAAs). To contribute to APCs maturation, this effect can be reinforced by OVs preloading with dedicated cargoes (e.g., GM-CSF gene as co-stimulatory treatment). OVs can promote intratumoral T-cell infiltration, for instance, by eliciting a type I interferon response. By inducing local acute inflammation, OVs can also reduce the impact of a suppressive TME. For a recent comprehensive review of the use for OVs as anti-cancer therapy, including a list of several clinical trials for OVs in cancer, I would refer the reader to Twumasi-Boateng et al. (77). Recently, Measles-based virotherapy has demonstrated synergistic activity with anti-PD-1 therapy in GBM treatment in a syngeneic C57BL/6 GL261 model (78). In a similar setting, intravenous delivery of GM-CSF/reovirus also showed synergistic activity with anti-PD-1. Moreover, intravenous reovirus delivery was also performed in human patients, and the viral payload was confirmed upon tumor resection, providing evidence of successful induction of the hallmarks of OVs activity (79). These examples are very important since checkpoint inhibition alone is insufficient to induce a response in GBM patients (80, 81). Preliminary clinical trials on OVs approaches are now completed and report on some positive indicators onto which more clinical and pre-clinical research should be designed) (82, 83), thereby underscoring the importance of animal models in testing strategies to awakening the immune system.

Thus, syngeneic models are becoming increasingly widespread. In addition to the mouse models, C6 glioma implantation in the fronto-parietal lobe of Whistar rats and 9L gliosarcoma in Fisher rats display features common to the human disease, such as proliferation, similar focal invasion as well as pseudopalisading necrosis surrounded by cells with great nuclear polymorphism (67). Interestingly, allogeneic 9L tumors in Wistar rats show a high infiltration of macrophages, microglia and CD4^+^/CD8^+^ T-cells that coexist with tumor lesions (84). This model has been successfully used to prove that dendritic cell therapy leads to enhanced tumor infiltration by CD4^+^/CD8^+^ T-cells and prolonged survival (85), and might represent a valid alternative to mouse models in the study of mechanisms of action for checkpoint inhibitors or therapeutic approaches targeting immune cells by other mechanisms, such as OVs.

### Homotypic and xenogeneic transplantation models

Traditionally, transplantation-based models served the purpose of testing the genetic dependence of tumor cells on individual genes or pathways (Table 2).

**Table 2:** Transplantation models for glioma.

**Tumor model**	**Origin**	**Transplantation mode**	**recipient animal**	**Transplantation type**	**Selected conclusion(s)**	**References**
GL261	3-methylcholanthrene into C57BL/6 mice	ic	C57BL/6	Syngeneic	*HIF1A* pattern of expression matches human GBM	Zagzag et al., (86)
9L glioma cells	N-nitrosomethylurea-induced tumor in Wistar rats	ic	Wistar rats	Syngeneic	Well-defined tumor mass but adaptive immunological response starting after 14 days (CD4^−^ T- and CD8^−^ T-cells)	Stojiljkovic et al. (84)
C6	methylnitrosourea (MNU)-induced tumor in Wistar rats	ic	Sprague-Dawley rats	Homotypic	High take rate for secondary tumors formed from C6 migrated in the contralateral hemisphere in primary passage	Chicoine et al., (87)
*E6/E7/hTERT/HRAS^*V*12^*	Immortalization of Human Astrocytes	ic	Rnu/Rnu rats	Xenogeneic	*RAS* and *TERT* activation and loss of both p53 and pRb pathways are required for astrocyte transformation	Sonoda et al. (88)
*EGFRvIII; INK4A/ARF^−/^*^−^	C57BL/6 NSCs or Astrocytes	ic	SCID mice	Homotypic	NSCs and astrocytes can both give rise to gliomagenesis with EGFR activation and *INK4A/ARF* loss	Bachoo et al. (89)
Patient-derived GBM	Human GBM	ic	NOD-SCID mice	Xenogeneic	CD133^+^ cells propagate brain tumors with higher efficience than CD133^−^	Singh et al. (90)
U87 and U251	Human glioma	ic, sc	SCID	Xenogeneic	Orthotopic implantation is superior to subcutaneous one in terms of imposing *in vivo*-specific gene expression	Camphausen et al. (91)
Patient-derived GBM	Human GBM	ic	Balb/c Nude	Xenogeneic	*in vitro* irradiation enhances tumorigeneicity of CD133^+^ GSCs	Bao et al. (92)
Patient-derived GBM	Human GBM	ic	SCID	Xenogeneic	GBM cells grown in NSC media better represent patients' histology	Lee et al. (93)
GL261	3-methylcholanthrene into C57BL/6 mice	ic	C57BL/6	Syngeneic	DC loaded with lysates from glioma cells propagated as GSCs confer more robust vaccination	Pellegatta et al. (73)
*EGFRvII*I; *INK4A/ARF*^−/−^; *EGFRvIII; INK4A/ARF*^−/−^; *BMI1*^−/−^	FVB NSCs or Astrocytes	ic	FVB or Balb/c nude	Homotypic	*BMI1* presence determines tumor growth rate, grading and phenotype.	Bruggeman et al. (94)
Patient-derived GBM	Human GBM	ic	NOD-SCID mice	Xenogeneic	Adherent cell lines preserve a more homogeneous undifferentiated profile	Pollard et al. (95)
9L glioma cells	N-nitrosomethylurea-induced tumor in Wistar rats	ic	Wistar rats	Syngeneic	Dendritic Cells loaded with tumor antigens induce intratumoral infiltration of CD8^+^ and CD4^+^ T-cells in a rat glioma	Liau et al. (85)
Patient-derived GBM	Human GBM	ic	NOD-SCID mice	Xenogeneic	CD44^+^ Neurosphere cells propagate brain tumors with higher efficience than CD44^−^	Anido et al. (96)
Patient-derived GBM	Human GBM	ic	CD1 nude	Xenogeneic	GBM Neurosphere cells propagate brain tumors regardless of marker expression but with different kinetics	Chen et al. (97)
Patient-derived GBM	Human GBM	ic	NSG	Xenogeneic	Anti-CD47 immunotherapy polarizes tumor-associated macrophages and increases survival	Zhang et al. (98)
Patient-derived GBM	Human GBM	ic	NSG	Xenogeneic	Lineage hierarchy is dominant to genetic and epigenetic heterogeneity in GBM propagation under homeostasis and therapeutic pressure	Lan et al. (99)
GL261	3-methylcholanthrene into C57BL/6 mice	ic	C57BL/6	Syngeneic	Oncolytic Measles cooperate with anti-PD1 immunotherapy	Hardcastle et al., (78)
GL261	3-methylcholanthrene into C57BL/6 mice	ic	C57BL/6	Syngeneic	NKG2D CAR T-cells prolonged survival benefit in mice and immunological memory against glioma	Weiss et al. (70)
GL261	3-methylcholanthrene into C57BL/6 mice	ic	C57BL/6	Syngeneic	Intravenous-injected GM-CSF/reovirus-reovirus accesses brain tumors in mice and sensitize to anti-PD-1 therapy.	Samson et al. (79)

While C6 tumors bear alterations in key tumor suppressors, such as the *Cdkn2a* (*Ink4a/Arf*) and *Pten*, in syngeneic as well as allogeneic transplantations, the transformation of astrocytes and neural stem cells derived from tumor suppressor mutant animals provides higher control of the tumor genotype (94), and represents a better setting for target discovery and validation (100).

A transplantation model based on immortalized astrocytes was instrumental in demonstrating that the loss of both the p53 and pRb pathways and gain of MAPK signaling and TERT-dependent telomeres protection are critical components in gliomagenesis (88). In the mouse, the minimal combination of the *Ink4a/Arf* locus deletion (i.e., p53 and pRb pathway inactivation) and constitutive EGFR activation by a glioma-specific mutant (i.e., MAPK activation) is dominant over the cell of origin (89). These seminal discoveries anticipated the demonstration that telomere protection can be reactivated by multiple means. TERT is reactivated, for example, in patient-derived glioma cells propagated under neural stem cell conditions, suggesting that upstream signaling can suffice (93). Moreover, there also exists a mechanism for the alternative lengthening of telomeres in GBM patients (101). Hence, while the control of telomere integrity is critical to gliomagenesis, TERT overexpression in itself may be a dispensable genetic manipulation in the process of glioma modeling in the mouse, despite promoter mutations define specific entities in human gliomas (63, 64).

Despite the fact that transplantation-induced stress can impart clonal expansion and affect gene expression, orthotopic transplantation should be considered the best approximation for a transplantation setting. In fact, subcutaneous and orthotopic growth impart very different transcriptional responses to the glioma cells' *in vivo* gene expression profile and response to treatment (91, 102). Consequently, orthotopic models for glioma also played a major role in the quest for potential epigenetic anti-cancer targets. The Polycomb group (PcG) gene *Bmi1* was shown to have oncogenic functions in gliomagenesis as a negative regulator of the *Ink4a/Arf* tumor suppressor as well as *Ink4a/Arf*-independent functions, as found in both mouse and human cells (94, 103). Likewise, the Polycomb repressive complex enzyme EZH2 also appeared to be required for glioma cell survival and proliferation in grafting experiments (104) and might be a good target reinforcing the adjuvant chemotherapeutic agent Temozolomide (99), even though context-dependent effects in the opposite direction were observed (105). Considering that Polycomb proteins contribute to coordinate the transcriptional response to converging pathways (100), the differential response to Polycomb inhibition may reflect the contribution of context-specific stress pathways within individual experimental settings. Alternatively, or in parallel, multiple cell populations composing the intra-tumoral mass may be differentially sensitive to Polycomb inhibitors (106), and their relative abundance may determine the overall response to the single agent.

To date, the genetic pathways that have been shown to prominently contribute to glioma growth in orthotopic mouse models have been largely validated by genomic studies in human patients (107), whereas targeting the epigenetic machinery as anti-cancer strategy still awaits the identification of effective combinations to account for compensatory mechanisms including adaptive responses and intra-tumoral heterogeneity.

Dissecting the tumorigenic potential of individual cellular populations that reflect this heterogeneity have also largely relied on the use of orthotopic models (Table 2). Following pioneering studies in leukemia (108), Dirks and colleagues demonstrated that tumor initiating ability in Non-obese diabetic, severe combined immunodeficient (NOD-SCID) mice is restricted to a subset of brain tumor cells (90). These cells in human GBM could be prospectively isolated using CD133 as surface marker (90). The ability to form neurospheres *in vitro* (109), and to withstand ionizing radiation (92) are critical biological features associated with “cancer stem-like cells” from high-grade gliomas. Surface markers, such as CD15 and CD44 have also been used to positively enrich for brain tumor initiating cells (96, 110). However, systematic comparisons of orthotopic grafts generated in CD1 Nude mice using glioma cell populations with different profiles of surface markers revealed that the non-enriched population also has tumor-initiating ability, with delayed growth kinetics (97). The difference for heterogeneous tumors, such as GBM may reflect the trans-differentiation ability of tumor cells into a wide range of lineages (111–114). Rather, the neural stem cell growth conditions of primary GBM cells proved to be a critical determinant in how accurately the orthotopic grafts resembled the patients' tumors (93, 95, 115). Collectively, these studies made enormous contributions to uncovering the cellular components of intra-tumor heterogeneity and highlighted the importance of orthotopic xenograft models.

Xenotransplantation is becoming increasingly the setting of choice for high-throughput target discovery and validation, a research area that recently evolved around the use of orthotopic models for cancer. Several pioneering studies in various allograft transplantation models have highlighted the importance of genetic screens in relevant physiological contexts (116–118). To cope with the numerical limitations imposed by the *in vivo* setting (119), genetic screens using RNAi in gliomagenesis (100, 120) were performed in immunocompromised animals with small libraries, or a genome-wide CRISPR was performed first *in vitro* and followed later by a parallel *in vivo*/*in vitro* validation screen (121).

As inferred by these studies, the use of immunocompromised recipient animals remains an invaluable tool in investigating cell autonomous mechanisms in human cancer cells *in vivo* and the choice of cellular models is therefore critical. Tumor models using established tumor cell lines have enormously contributed to our knowledge of tumor biology but are increasingly being dismissed in gliomagenesis experiments (122). The state-of-the-art in the field of brain tumors is to propagate tumor cells under conditions supporting non-transformed the *in vitro* self-renewal of neural stem cells (93). Limitations to this approach include that: (i) *ex vivo* propagation remains anchored to the assumption that signaling, supplements and environmental conditions are known and can be delivered homogeneously, (ii) some low-grade tumors as well as specific genotypes systematically drop out in these conditions (123). Nonetheless, sophisticated *ex vivo* culture conditions represent the best approximation to preserve tumor identity while enabling experimental manipulations or *in vitro* screening endeavors (115). Indeed, when compared to patients' biopsies, conventional glioma cell lines fall short of representing patients' molecular profiles (53).

### Patient-derived xenografts models

An elegant approach to bypass cultures while focusing on patient-oriented modeling is to generate Patient-derived xenografts (PDX) or Avatar models, created by patients' biopsies without applying *ex vivo* culturing prior to transplantation (124). PDX models propagate the complex cellular and genetic heterogeneity of the cell surviving in the host animal, and are therefore capable of modeling responses to standard, targeted or combination therapies without forcing assumptions on the tissue sample. A variant to preserving patients' biopsy tissue structure is dissecting the tissue prior to the transplantation of live cells. This is usually exploited to bypass the low take rate for some tumors, a drawback affecting low-grade or high complexity tumors. A similar approach is used to perform limited experimental manipulations followed by serial transplantation (99).

There is a general consensus that PDX models maintain some level of concordance between patients and PDX responses to therapy (125, 126). This includes GBM, which partly preserves molecular profiles principles during xenotransplantation (53, 127). Importantly, however, the response to anti-cancer treatment largely depends on the tumor cell genotype at the time of the treatment (127). The latter piece of evidence is relevant in that both *ex vivo* cellular passaging and PDX intrinsically suffer a drift toward genomic instability. In a recent large-scale study, the dynamics of copy number alterations (CNAs) in 1110 patient-derived samples of different cancer types during multiple rounds of *in vivo* propagation have been reported. A high rate of CNAs was observed in xenografts that artificially drift away from the human counterpart. For instance, glioblastoma patients acquire extra copies of chromosome 7 during tumor evolution, whereas PDX propagation in mice results in a loss of these extra copies. While the genetic drift is not surprising for malignant gliomas given their near-complete deficiency in DNA damage checkpoint control (128), these results raise awareness of the limitations associated with the use of PDX as patients' avatars to evaluate their responses to any given therapy and call for integrating this resource with more stable models (129). It is also critical to realize that responses to therapies in PDX models will be affected by the intra-cellular heterogeneity of the transplanted tumor. Using an elegant cellular barcoding strategy, the Dirks lab has recently demonstrated that a number of different cancer cells within a tumor can contribute to its homeostasis (99). Strikingly, however, this number can change from one tumor to the next, thereby affecting the reproducibility of hypothesis testing or target discovery and validation experiments.

In immune-oncology, transplantation models are the preferred choice when testing the impact of innate immune checkpoint inhibition and CAR T-cells. For instance, disruption of the CD47/SIRPα signaling affects leukemia, glioma, melanoma and hepatocellular carcinoma growth as xenografts (98, 130–132). The efficacy of GD2-CAR T-cell against glioma cells was pre-clinically tested in NSG mice until the onset of graft-vs-host disease symptoms (GvHD; ~4 weeks), and GD2-CAR T-cells are currently tested in several clinical trials (among others, see NCT03252171).

These studies collectively highlight the continuing process of addressing the intrinsic challenges associated with transplantation models and expanding these to advanced pre-clinical settings, anticipating that this experimental system will retain its leading role in experimental medicine.

### Next-generation autochthonous models

**Figure 1:**
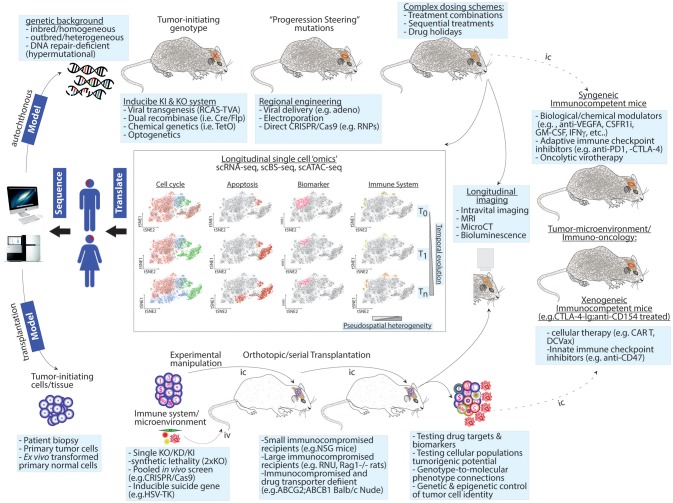
Next-generation modeling of human cancers: longitudinal single-cell “omics” in autochthonous and transplantation-based models. Upper panel: genetic drivers of human cancer are combined with a genetic background of choice to give rise to homogenous, heterogeneous, or hypermutational tumors. Genetic engineering can use independent genetic and/or chemical switches to permit tissue-specific and temporal control. To increase tumor heterogeneity or test the contribution of mutations identified at recurrence upon tumor formation, intratumor injections of “steering” mutations using viral or other means can be used. These sophisticated cancer GEMMs permit testing complex dosing treatments. Lower panel: to test genetic dependences or perform genetic screens, patient-derived or *ex vivo* transformed tumor-initiating cells are genetically modified and/or barcoded and then transplanted into recipient animals. The host background can be chosen to favor tumor take and/or drug penetration. In this setting, a co-grafting of adaptive or innate immune cells or other microenvironment players (e.g., endothelial cells, pericytes, etc.) or serial transplantation can be implemented to study non-cell autonomous mechanisms and to exacerbate competition among cancer cells. Heterogeneity increases as a function of time and intra-tumor complexity. Both experimental models can be exploited in longitudinal follow-ups using live imaging or single cell “omics” (central panels). The latter approach can simultaneously generate spatiotemporal information on changes in cell cycle progression, apoptosis, cell fate decisions/microenvironment activation (i.e., biomarker variations) and immune cell composition. Right panel: single cell preps from GEMMs or orthotopic transplantation models can be transplanted (dashed line) in the indicated immunocompetent animals thereby creating a syngeneic/xenogeneic models, respectively. Potential applications for these models are indicated. T0, T1, and Tn = time points for longitudinal analyzes; pseudospatial complexity refers to the output of tSNE maps as a surrogate for spatial information. Iv, intravenous; Ic, intracranial.

In mice, the CRISPR revolution will make the generation of complex animal models increasingly easier, faster and affordable (Figure 1).

Tumor heterogeneity and evolution is a major feature of human cancers (133, 134), and these features have proven hard to model autochthonously. One important consideration for the design of future, next-generation mouse models is their genetic background. Most mouse strains are inbred and represent a great advantage in research to unravel specific targets or mechanisms in a certain type of cancer (135). Yet inbred strains are far from the real diverse setting of disease development in humans, and the genetic background of animals used as pre-clinical cancer models in research is critical to the outcome of the study. In gliomagenesis, *Nf1* and *p53* deletion leads to a tumor grading ranging from low-grade astrocytoma to GBM depending on the distinct mouse strain (29). Likewise, *p53* heterozygosity leads to a spontaneous formation of mammary tumors in BALB/c mice but not in C57BL/6J (136), suggesting a distinct pro-tumorigenic genetic background in BALB/c mice. Moreover, the RF/J strain displays a high incidence of cancer owing to missense mutations in DNA damage repair and cancer-associated genes and may become the model of choice for genetically hypermutated tumors, such as smoking-driven lung cancer, UV-driven melanomas and DNA repair-deficient colon cancer. The choice of the background for future GEMMs may be driven by the clinical history of individual diseases and may exploit the Collaborative Cross Consortium 2012 benchmarking of several inbred mouse strains (137). In turn, this may also facilitate testing the contribution of natural variation and DNA repair in tumorigenesis and responses to treatment. This appears to be particularly important if one considers that genetic variation may not only affect tumor-intrinsic, but also and even more likely, the tumor-immune phenotype.

The recent conclusion of a colossal genomic investigation in almost 10,000 genomes spanning 33 cancer types by the Cancer Genome Atlas (TCGA) has provided an enormous dataset of cancer driver genes (138). *In vivo* validation and hypotheses testing follow-ups are lagging behind. Simultaneously testing dozens of putative tumor suppressors within a native and immunocompetent microenvironment is now possible. In a medium throughput *in vivo* screening in gliomagenesis using the stereotactic injection of a sgRNA adenoviral library for CRISPR/Cas9 mediated knock-outs, the Sidi Chen lab reported on ability for PanCancer-defined significantly mutated genes to initiate brain tumor formation (50). The combination of regional viral delivery and GFAP-Cre conditional activation of Cas9 and GFP restricted the screen to a subset of glial cells. This type of screen follows the direct RNAi delivery pioneered by the Zender lab in hepatocellular carcinoma (139). While the inability of AAV to genetically integrate into the host genome requires extensive target sequencing as direct readout, compared to earlier studies, this provided a significant higher throughput in screening for combinatorial signaling dependencies during gliomagenesis (38, 43). It also represented a more physiological setting for tumor suppressor discovery than transplantation models previously used in similar endeavors (100). Considering that adult GBM is a disease that often develops over several years and goes through regional and temporal evolution (140, 141), future studies along these lines may benefit from integrating more sophisticated, spatiotemporally precise forms of editing. Examples of the sequential delivery of cancer driver mutations exploiting classic genetic recombination techniques were previously reported (142). To enable further spatiotemporal control, biochemical, chemical or optogenetic control of tumor progression may be employed. This would be particularly important in order to better mimic disease initiation and progression (Figure 1).

An enhanced control of tumor progression using advanced genetic engineering methods may permit a validation of driver and passenger mutations as modifiers of tumor progression and responses to therapy or testing advanced genetics paradigms, such as genetic essentiality (22). To this end, while direct intracranial injection of RNP complexes has not yet been exploited in models of glioblastoma, it has been successfully applied to genome editing in the mouse brain (143). During brain tumor exposure to multimodal therapy, additional aberrations in core GBM driver pathways are acquired. Combining longitudinal intravital imaging and topical delivery of RNPs or *in situ* electroporation-inducing genetic engineering may help in elucidating the contribution of genetics to disease recurrence. Genetic alterations identified as critical regulators of tumor evolution could be defined as “steering” mutations, and would become an integral part of sequential modeling. In GBM modeling, two examples of such mutations may be *Nf1* and *Msh6*, both of which are associated with recurrent tumors and TMZ-induced hypermutations (144, 145). Importantly, this approach would also permit the functional dissection of cell-autonomous mechanisms, such as tumor microtube formation (146), as well as non-cell autonomous processes, such as vessel dysmorphia (147).

Unpredictable adaptive responses to anti-cancer treatments are a hallmark of solid tumors. Cancer cells can evade chemotherapy by acquiring additional mutations, switching to a state of negligible growth, by activating survival pathways through changes in cell identity, and by other means. In modeling human cancer in mice, one should account for possible species-specific tumor genotype drifts that would not be representative of the patients'. Thus, next-generation GEM models that better represent primary tumors and their evolution at a genetic level (as depicted above) will be invaluable tools for testing complex chemotherapy-dosing schemes (Figure 1).

Longitudinal studies in humans are revealing oncogenes dominant in recurrence. In GBM, mutations in *NF1* and *PI3KCA* appear to drive disease progression (145, 148, 149), and may be temporally controlled to mimic disease progression. In such models, complex sequential drug combinations may be tested. For instance, switching off NF1 or PI3KCA, which reinforce the RAS pathway and confer sensitivity to a combination of BRD4 and MEK inhibition (150). A neoadjuvant-like multimodal therapy followed by targeted drugs, would be prototypic examples of testing complex targeting of acquired mutations.

Metronomic chemotherapy and planned drug holidays are complex dosing schemes that aim at reversing drug tolerance. In GBM, tumor cells evade adjuvant Temozolomide using MGMT reversal and cell cycle restriction. A Temozolomide holiday may be therefore alternated with targeted therapies, such as blood-brain-barrier penetrant PI3K inhibitors (151).

An emerging paradigm in cancer biology is to drive cancer cells to acquire specific addictions and then targeting such addictions with drugs. This approach may be important in exploiting metabolic (152, 153) as well as epigenetic targets (154), or targeting collateral lethality associated with acquired resistance mechanisms [(155) and our unpublished data]. This includes also extrinsic mechanisms, such as those targeted by adaptive immune checkpoint inhibitors. Indeed, studies in syngeneic models have shown promising results in a combination or neoadjuvant setting that induced PD-L1 expression (79, 156).

While complex dosing schemes have been so far directly tested during clinical trials, the combination of accurate GEM models and complex readouts should make it standard practice at the pre-clinical level to test whether low-dose or sequential treatments are equally or even more effective than drug combinations, thereby sparing patients from the side effects of the added toxicity and possibly enhancing tumors' response. In this regard, once the specie-specific differences are accounted for, the autochthonous models are well-positioned to provide the most physiological setting for tumor initiation and organismal response to treatments.

### Next-generation transplantation models

Transplantation models are currently developing on parallel research lines.

To avoid the complications of genetic drifts as well as patient-specific passenger mutations, PDX xenograft models are being complemented with *de novo* transforming human cells derived from normal tissue with relevant cancer mutations in the projected neoplasia. Transforming human astrocytes has been instrumental in formally testing the contributions of the main pathway required for efficient gliomagenesis in human cells. Recent work using human colon organoids has shown that combining an appropriate cell of origin and set of mutations is still insufficient to recapitulate some of the biological properties of the true tumors, such as metastasis (157). Future work in this direction should be aimed at precisely dissecting genotype-to-phenotype connections in human cancers using advanced genetic screening systems. In this regard, systematically transforming normal mouse and human cells to create next-generation avatar models is expected to help address some open questions. For instance, in numerous solid tumors, the role of copy number aberrations remains to be clarified. Whereas, human GBM is clearly a disease of copy number aberrations (158), studies in autochthonous models so far fall short of clarifying whether and how this feature contributes to the disease. Moreover, emphasis should be given to developing an understanding of molecular phenotype specifications for tumors including GBM in which there is limited evidence of genetically encoded subtypes. Overall, creating reliable and homogenous tumor-initiating cells *in vitro* may complement PDX-models and permit testing *ad hoc* biological questions as well as providing a more reproducible resource for target discovery and validation (Figure 1).

The recipient animals are a critical determinant of the success of transplantation models. The informative value of such models is dependent on both cell-intrinsic and non-cell autonomous components. On the one hand, achieving the highest grafting potential through the use of severely immunocompromised animals is the essence of xenotransplantation (159). On the other, the discovery of adaptive checkpoint inhibitors and the need to identify the basis for responses to immunotherapy pose additional challenges to modeling human tumors using xenotransplantation; one solution is to reinstate the adaptive immune system in host recipients. Freshly isolated peripheral bone marrow cells (PBMCs) or specific immune cell subpopulations can be co-grafted with human cancer cells subcutaneously in immune deficient animals (e.g., NSG/NOG). Alternatively, PBMCs can be parentally infused (i.p. or i.v.) after subcutaneous or orthotopic tumor transplantation has taken place. This approach revealed, for instance, the immunogenic effect of a Carcinoembryonic Antigen CD3 T-Cell Bispecific Antibody (CEA-TCB) in promoting the infiltration of xenografts as well as adaptive PD-L1 over-expression, thereby suggesting the feasibility of combining its administration with adaptive immune-checkpoint inhibitors (160). Whereas this setting is limited to short-term studies given the potential for severe GvHD, it also enabled assessments of the efficacy of immunotherapies and the impact of T-cell subpopulations (e.g., regulatory T-cells; T-regs) or antigen-presenting cells (APC) on the activation of cancer cells (161).

The GvHD is not only a complication for the recipient animals. Immune cells that suddenly face a full-blown tumor to which they were previously naïve face an acute pathophysiological stimulation reflecting the actual tumor. Ideally, future models will feature intact innate and adaptive immune systems trained to ignore the grafting phase and triggered by pathophysiological stimuli to transiently allow grafting. An elegant example of this approach in glioma models has been achieved by blocking the T-cell activation by APC through CTL4A-IgG (clinical name: Abatacept) and anti-CD154. Immune-tolerant mice generated by this approach developed GBM lesions and these affected survival with similar kinetics as immune-deficient animals (162). As an alternative in immune-deficient backgrounds, co-grafted immune cells may be pre-exposed to tumor cells (e.g., DC loading with tumor cells lysate or RNA), thereby more closely mirroring the gradual rise of tumors in humans. Moreover, some innate immunity players may require specific co-grafting schemes. For instance, microglia cells, the resident macrophages of the central nervous system, are generated during embryonic development (163). These cells are associated with the full specification of the Mesenchymal GBM subtype identity (164–166), and most likely with responses to treatment (167). This situation calls for a reliable source of human microglia whether tumor-isolated or *in vitro* immortalized to serve as co-grafting partners in transplantation models.

The genetic engineering of recipient animals can also be exploited to selectively impair the tumor microenvironment. To determine the role of drug penetration and reabsorption in brain tumor models, for instance, the drug transporters ABCB1 and ABCG2 were deleted not only in wild-type glioma models, but also in immunodeficient recipients in xenotransplantation models (168, 169).

In the neurosciences, the rat is the experimental and translational model of choice wherever possible. This species is considered superior for etiological, pathophysiological, pharmacological and behavioral studies (170). The discovery of culture conditions that facilitate the expansion of rat embryonic stem cells as well as the direct use of CRISPR during zygote formation may enable extending also to rats the generation of complex models for gliomas. This would allow extending the use of these animals from a limited pharmacodynamics setting to a fully-fledged advanced modeling system.

The future development of transplantation models at the levels of both tumor cells and recipient animals will enable more sophisticated experimental dissections of cell-intrinsic and non-cell autonomous mechanisms and a more effective platform for target discovery and validation.

### Outlooks and perspectives for single cell “omics”

Interrogating cell cycle responses, apoptosis and cell identity drifts at the level of single cells is a transformational change in that it permits assessing the effects of a compound and simultaneously to build hypotheses based on possible combinatorial treatments or sequential treatments (Figure 1).

Single-cell RNA sequencing has been significantly exploited in GBM to describe intratumor heterogeneity and the peculiarities of GBM subtypes and their microenvironments (164–166). This approach has also been instrumental in identifying genetic and transcriptional identities associated with tumor-specific biological properties, such as proximal and distal recurrence, infiltration and numbness to the fluorescence-guided probe for the resection of diseased tissue (5-Aminolevulinic acid, also known as 5-ALA) (149). DNA methylation is being currently implemented in clinical neuropathological practice for brain tumor classification (171). The availability of technologies enabling to simultaneously generate single cell RNA-seq (scRNA-seq) and bisulfite-converted DNA methylation (BS-seq) maps from the same cell (172) has a potential to allow the tracking of tumor responses to treatments. In turn, this will be a major driver in the development of targeted therapies in both autochthonous and transplantation models (Figure 1).

I envision that the combination of multiple autochthonous models in parallel and scRNA-seq and BS-seq may be exploited to evaluate the way the core tumor and in the infiltrating margins respond to individual treatments. Importantly, metabolic labeling of RNA *in vivo* now enables identifying faster and more accurately the adaptive transcriptional changes (173).

To bridge preclinical and clinical testing, longitudinal scRNA-seq in GEMMs may help assessing and improving adjuvant and second-line treatments. To some extent, autochthonous models of aggressive human cancers may well represent low Karnofsky performance score (KPS) patients or very hard-to-resect tumors, in that surgery is discouraged. In these cases, single cell profiling will be very informative in testing the consequences of standard approaches, such as radiation followed by adjuvant treatments (e.g., in GBM, Temozolomide, Bevacizumab) and provide benchmarks for new treatments. This matter has so far been restricted to advanced clinical trials in patients and may be now repositioned at the preclinical stage.

Exceptionally, single cell “omics” and autochthonous models may also help testing next-generation probes for optically guided surgery. 5-ALA is currently used in fluorescent-guided surgery to delineate tumor margins for resection, which is extremely important in preventing local recurrence in the brain parenchyma, where surgeons need to be conservative. Recent studies in GBM at the single-cell level suggest that 5-ALA appears to mark Mesenchymal subtype-specific GBM cells, leaving behind Proneural-subtyped cells (149). It will be important to test this in autochthonous models in which the two states can be modeled (56), and to compare 5-ALA to novel fluorescent probes. By resecting a tumor margin pre-labeled with such probes, single cell profiling will reveal the identity of each cell that retain or miss the labeling.

The xenogeneic transplantation setting is well-versed for testing intrinsic cellular responses to novel treatments and detecting adaptive resistance, notably in cases in which tumor perfusion is homogeneous. Indeed, the response to intracellular targets for drugs are best assessed in human cells when on-target and off-target effects need to be accounted for. Moreover, applying CRISPR screens to transplantation models using scRNA-seq readouts, such as CROP-seq (174) may improve the resolution of *in vivo* functional screens, thereby increasing their throughput. In the brain tumor setting, these were limited so far to a few dozen targets (100, 120), and CROP-seq may lead to more comprehensive screens without compromising the pathophysiology of the orthotopic transplantation setting (119). Importantly, even in the absence of functional perturbations, approaches like CROP-seq coupled with scRNA-seq will enable a next-generation of cellular barcoding experiments to trace *in vivo* tumor homeostasis and responses to treatments (99). In this area, PDX models should more systematically be complemented with *de novo* transforming human cells and with models better representing tumor molecular profiles.

Harnessing the therapeutic potential of the TME and immuno-oncology will significantly benefit of building experimental consensus within the community. In particular, it is critical to define the appropriateness of any given model in the assessment of the response to treatment of established tumors. Differences between animal models and humans specifically involve protein-coding genes and cis-regulatory DNA due to specie-specific adaptive selection, notably those controlling immunity (175, 176). Moreover, the consistency in the response of human cells to mouse supplements and *vice versa* (e.g., growth factors, cytokines, etc.) are largely anecdotal. Nevertheless, whether GEMMs, syngeneic or xenogeneic models based on orthotopic transplantation may be used for testing of TME- and immune-therapies should be systematically assessed when appropriate readouts can be faithfully reproduced in both species. This has been possible, for instance, in the quest for identifying antigen-specific TCRs. In this case, comparing immune-deficient mice reconstituted with human hematopoietic progenitors (i.e., humanized mice) and mice transgenic for the human TCRα and TCRβ loci returned similar results (177).

Despite the acknowledged pathophysiological relevance of GEMMs, syngeneic models based on established cell lines have been preferred in investigating TME- and immune-therapies (Table 2). To exploit the CRISPR/Cas9 potential in the future models, I anticipate that primary tumor cells from sophisticated GEMM models may soon replace cell lines in a secondary syngeneic orthotopic setting. This way, one could harness the genetic and spatiotemporal controls in an immune-tolerant setting (Figure 1, right panel). This approach would be the ideal development for the preclinical dissection of intrinsic, acquired and non-cell autonomous resistance mechanisms. In the example provided by the combinatorial or neoadjuvant OVs treatment, syngeneic models may be used to uncover the mechanism conferring residual resistance, which is indicated by the incomplete penetrance of the sensitization to checkpoint inhibitors (79, 156). Longitudinal scRNA-seq may reveal the optimal timing for starting the immune-checkpoint inhibition and whether cell intrinsic or other cells from the microenvironment are contributing to or preventing a fully penetrant response.

Whereas testing immunotherapy strategies in immunodeficient animals appears counterintuitive, the use of NSG mice emerges as the mainstream strain for studies involving sophisticated reagents eventually used in clinical trials. These include systems for T-cells engineering (178, 179), and innate immune checkpoint inhibitors (98, 130–132). While retaining a leading position in these experiments, a foreseeable evolution of this system will involve improving the recipient animals, including transplanting human cells into highly immunodeficient rats.

Transformative technologies, such as CRISPR/Cas9 and single-cell genomics have opened new avenues in the study of tumor biology. Here I propose that the longitudinal dissection of tumor responses in animal models can capitalize on single cell genomics approaches. The specific examples I have discussed here are cases that could clearly improve our current understanding of human cancers and their responses to treatment. Combining genetic engineering and single-cell genomics with the individual strengths of GEM and transplantation models is bringing about novel next-generation platforms for understanding tumor biology and for target discovery and validation.

## Author contributions

The author confirms being the sole contributor of this work and has approved it for publication.

### Conflict of interest statement

The author declares that the research was conducted in the absence of any commercial or financial relationships that could be construed as a potential conflict of interest.
